# Tracking Devices for Pets: Health Risk Assessment for Exposure to Radiofrequency Electromagnetic Fields †

**DOI:** 10.3390/ani11092721

**Published:** 2021-09-17

**Authors:** Judith Klune, Christine Arhant, Ines Windschnurer, Veronika Heizmann, Günther Schauberger

**Affiliations:** 1 WG Environmental Health, Department of Biomedical Sciences, University of Veterinary Medicine Vienna, Veterinärplatz 1, A-1210 Vienna, Austria; Judith.Klune@vetmeduni.ac.at; 2 Institute of Animal Welfare Science, Department for Farm Animals and Veterinary Public Health, University of Veterinary Medicine Vienna, Veterinärplatz 1, A-1210 Vienna, Austria; Christine.Arhant@vetmeduni.ac.at (C.A.); Ines.Windschnurer@vetmeduni.ac.at (I.W.); Veronika.Heizmann@vetmeduni.ac.at (V.H.)

**Keywords:** tracking device, health risk, exposure, radiofrequency electromagnetic fields, lost pets, reunion, collar

## Abstract

**Simple Summary:**

To increase the probability of reunions occurring between owners and lost pets, tracking devices are applied to pets. The pet’s position is determined by satellites (e.g., GPS) and transmitted by radio frequencies (RFs) to a mobile phone. In this study, the health risks from exposure to radio frequencies emitted by radios, TVs, mobile networks, indoor devices (e.g., WLAN, Bluetooth), mobile phones, and in the use of such tracking devices were investigated. The radiation exposure was found to be well below international limit values, which means that adverse health effects are unlikely to occur. The risk of high exposure of pets is mainly caused by indoor RF-emitting devices, such as WLAN devices. This exposure can be limited through a reduction in the exposure time and an increase in the distance between the animal and the RF-emitting device. Even though the exposure of pets to total radiofrequency electromagnetic field (RF-EMF) levels was found to be below the limit values—and, therefore, not a health risk—recommendations are given for the use of tracking devices and to limit the exposure to indoor devices.

**Abstract:**

Every year, approximately 3% of cats and dogs are lost. In addition to passive methods for identifying pets, radiofrequency tracking devices (TDs) are available. These TDs can track a pet’s geographic position, which is transmitted by radio frequencies. The health risk to the animals from continuous exposure to radiofrequency electromagnetic fields (RF-EMFs) was reviewed. Fourteen out of twenty-one commercially available TDs use 2G, 3G, or 4G mobile networks, and the others work with public frequencies, WLAN, Bluetooth, etc. The exposure of pets to RF-EMFs was assessed, including ambient exposure (radios, TVs, and base stations of mobile networks), exposure from indoor devices (DECT, WLAN, Bluetooth, etc.), and the exposure from TDs. The exposure levels of the three areas were found to be distinctly below the International Commission on Non-Ionising Radiation Protection (ICNIRP) reference levels, which assure far-reaching protection from adverse health effects. The highest uncertainty regarding the exposure of pets was related to that caused by indoor RF-emitting devices using WLAN and DECT. This exposure can be limited considerably through a reduction in the exposure time and an increase in the distance between the animal and the RF-emitting device. Even though the total RF-EMF exposure level experienced by pets was found to be below the reference limits, recommendations were derived to reduce potential risks from exposure to TDs and indoor devices.

## 1. Introduction

Over any period of five years, approximately 15% of cat and dog owners lose their pets. Many animals cannot be identified by those that find them, which means that only 38% of these finders are able to reunite the pets with their owners, with dogs much more likely to be returned to their owners (46%) than cats (3%) [1]. Vienna’s largest animal shelter, Tierquartier Wien, counted over 800 stray pets in 2020, of which approximately 70% of the dogs and 30% of the cats were successfully reunited with their owners. In total, 75% of these pets were already implanted with radiofrequency identification (RFID) transponders to increase the possibility of reunion. However, only half of the chipped animals were registered and just 50% of them were correctly registered by their actual owner. A correct registration increases the probability of reunion. As there are still many pets that are lost and cannot be returned, pet owners can use various methods to increase the chances of reunion with their cat or dog. One approach involves the use of passive identification methods through which the animal and the owner can be identified. This means that the finder has to contact an animal shelter or a veterinarian to identify the animal. Another method involves the use of active TDs, which can derive the geographic position of an animal by using global navigation satellite systems (GNSSs) (e.g., the Global Positioning System (GPS) or the European system GALILEO). The position is transmitted by radio frequencies, which provide the owner with a way to find his/her cat or dog on their own by identifying their pet’s exact location.

Currently, the following passive identification methods are in use: (1) ID tags, visibly worn by the animals; (2) RFID transponders, which are invisible and need a scanning device to read out data; and (3) tattooing of the animals with visible identification. Methods (2) and (3) require a shared database for the identification data to be stored. In general, identification can be conducted by animal shelters, veterinarians, or people who have access to the database and an RFID reading device.

The simplest identification method is to use an ID tag, which is mounted on a collar (for cats and dogs). The advantage of this method is the visibility of the ID tag for the finder. ID tags are inexpensive and easy to obtain and use. However, ID tags can get lost. RFID transponders are a permanent method for pet identification, as they include a unique identification that is enclosed in an inert glass capsule. This method is most commonly used for dogs, cats, and horses, where the chip is subcutaneously implanted laterally in the neck (Europe) or between the shoulder blades (UK, USA) of the animal [2,3]. The implications of using RFID transponders to identify lost dogs or cats have been investigated in Australia and the US. In Australia, the proportion of chipped cats reclaimed by owners was 33–61% compared to only 5% for unchipped cats [2]. Lord et al. [4] reported that the reunification frequency for stray dogs was 21.9%, whereas the frequency for stray dogs with RFID transponders was 52.2%. The frequency of recovery was 1.8% for stray cats, whereas 38.5% of chipped cats were reclaimed. In Austria, on average, only 5% of cats that enter a shelter can be returned to their owners, in contrast to 28% of dogs [5]. When the owner’s information was stored in a registry or in an animal shelter database, the reunification success was increased by a factor of 4.9 for cats and 8.7 for dogs compared to owners who were not registered [4]. The advantage of visible identification methods is stated as follows by the American Association of Feline Practitioners [6]: *“Visual identification methods such as a collar with a tag provide an immediate source of identification, which anyone can use to contact the cat owner at any time”*. Although passive identification methods increase the frequency of unification [6], active systems, such as TDs, possibly have an even greater reunion success rate. This seems to be due to their advantage of being able to instantaneously identify the geographical location of the stray animal.

This report includes an overview of commercially available TDs in order to identify the technical features that influence animals’ RF-EMF exposure. The exposure levels were assessed not only for TDs but also for the emissions of other RF sources. The biological effects and limit values for RF-EMFs and the risk to the animals were evaluated. Based on the risk assessment, recommendations were derived to reduce the overall exposure levels that animals experience.

## 2. Tracking Devices for Animals

### 2.1. Technology

**Figure 1:**
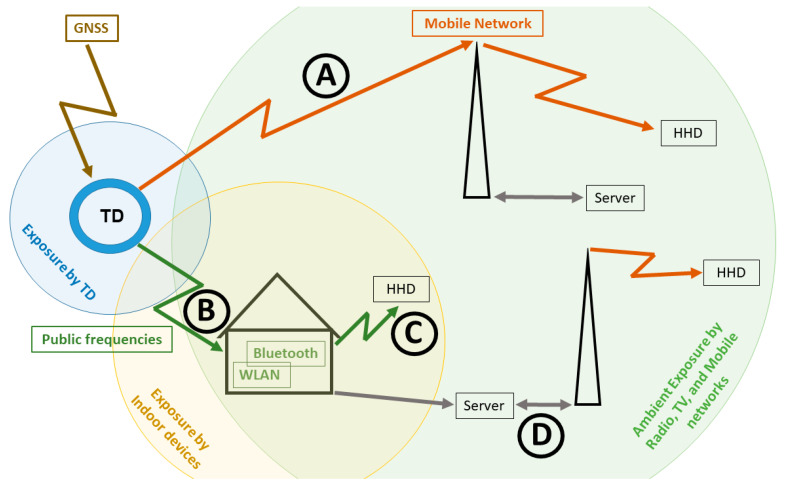
Schematic diagram showing the pathways of the global navigation satellite system (GNSS) signal used by the tracking device (TD) and the transfer of the positioning data via a mobile network to a handheld device (HHD) (pathway **A**) or via licence-exempt frequencies (WLAN, Bluetooth) to a base station at home (pathway **B**). The data are then transferred in the near field by WLAN or Bluetooth (pathway **C**) to an HDD or via the Internet to a mobile phone (pathway **D**). The degree of exposure of pets to RF-EMF by TDs, indoor devices, and radios, televisions, and the base stations of mobile networks (ambient exposure) is shown by coloured circles.

The review focused on TDs that are used for pets. Systems to track wild animals were not included in the review. The first system is a ground-based system that uses an RF transmitter with a license-exempt frequency that is allocated for this purpose. The position of the animal can only be detected by a handheld loop antenna, which indicates the direction of the animal, whereas the distance can only be assessed by the signal strength, shown by a modification of the tone pitch of the signal and by colours. This technology works in a range of approximately 120 m. The second system uses a global navigation satellite system (GNSS) to determine the animal’s geographical position. The position is transmitted in various ways to handheld devices (HHDs), such as mobile phones or tablets. The tracking data can be transmitted by using a mobile network over distances without limitation (Figure 1, pathway A). For close environments, a wireless local area network (WLAN) or Bluetooth is used to broadcast the tracking position to a base station (pathway B) and afterwards directly to an HHD (Figure 1, pathway C) or it is transmitted via the internet and a mobile network to an HHD (Figure 1, pathway D). Data transfer using a mobile network requires a contract with a provider (by using a prepaid SIM card or any tariff with at least 100 MB), but costs could increase if roaming fees have to be paid abroad.

The GNSS offered by the USA is called the Global Positioning System (GPS), that offered by the European Union is called GALILEO, the system offered by Russia is called GLONASS, and that offered by China is called BEIDOU [7]. In many cases, the embedded electronic module in a TD can derive the position using more than one of these systems. The positioning accuracy varies depending on time-specific satellite coverage and the topography surrounding the pet. If the GNSS signal is too weak to determine the position, a rough estimation can be achieved by triangulating the local positions using several base stations of the mobile network, depending on the radius between the mobile network cells. This estimation of the position, which is called a location-based service (LBS), is less accurate than GNSS. Moreover, Bluetooth or WLAN might also be used by some devices if the GNSS signal is not available indoors to help determine the position in the surroundings.

In addition to the geographic coordinates of the position, other information, such as environmental parameters (e.g., temperature) and the parameters describing the physical activities of the animals, can be transmitted [8].

### 2.2. Overview of Tracking Devices for Pets and Horses

Twenty-one TDs for pets (cats and dogs) and horses were analysed according to their technical features, for instance their technical usage characteristics such as battery lifetime, dimensions, weight, and functionality. A special focus was placed on the technology used to transmit the geographic positions of the animals by radio frequencies in order to assess the exposure. Moreover, the animal species for which the TDs were designed were identified, and the customers incurred both one-off expenses and running expenses. The information used for this survey was collected from the companies’ web pages and the TDs’ operation manuals and by submitting requests directly to the companies. The technical features are summarised in Table 1.

Seven out of twenty-one of the TDs are designed for dogs, four for cats, eight for dogs as well as for cats, and two are offered for other pets above a specific weight. Additionally, two of the TDs work for horses. The TDs are fixed to the pets’ collar or harness. Nineteen out of twenty-one of the TDs are able to report the locations of tracked animals to a server and use an app that allows the owner to follow the animal’s position on a map (Figure 1, pathway A or B + D). This mobile technology provides a user-friendly capability and the possibility for diverse features, tools, and system applications, such as the Google Maps mapping service-based system.

Twenty of the TDs derive the geographic position using GNSSs. Fourteen of the TDs use a mobile network to transmit the tracking data, which means that there are no restrictions on the distance of the pet (Figure 1, pathway A). Of these, eleven use the 2G protocol; only three use 3G or 4G transfer protocols. For areas in which no mobile networks are available, two TDs use the frequencies of 170 MHz and 915 MHz to transmit the tracking data in the far field to an HHD (*Garmin* and *PetFon with PetFon Mash*) or, as in the case of *PetFon*, which transmits the data to the *PetFon Mash*, to a base station; both work within a range of approximately 10 km.

Four TDs use licence-exempt frequencies, which are allocated for this purpose and indicate a base station with a much lower operating distance. This technique is applied for pets near the home, as the range is only a few kilometres (*Findster, PetTracer, PetFon without PetFon Mash*), and for horses to survey activities on the pasture (*Hoofstep*).

TDs that do not use mobile networks have no subscription plan and usually no monthly fees (except *PetTracer*). Additionally, six TDs use near-field (Bluetooth and WLAN) and far-field technologies combined (Figure 1, pathway B).

The last option involves the use of a direction-finding transmitter and a loop antenna to locate the animal. This technology is used by three TDs, of which two are for cats only and one is for both cats and dogs (*PetTracer, Cat-Control, Kippy). Cat-Control* only uses this technology and is therefore the only TD that does not use a GNSS. Moreover, a mobile app on an HHD is not available in this case. For cats, a recent study showed that the majority of cats have rather small home ranges (3.6 ± 5.6 ha) and, approximately and on average, travel only 100 m away from their owners homes [9], with very few cats having large home ranges of up to 8.6 km^2^. Several characteristics, such as sex, neuter status, age, and the environment (urban vs. rural), can influence roaming behaviour [10], with young intact males in rural environments having the largest home ranges [9,10].

Most of the TDs offer the possibility of establishing digital fences/geofences, which surround the areas within which the animals are allowed to move, and the devices have a standby mode that can be used with longer transmission intervals. Overstepping these digital fences causes the TD to become activated, which results in an alert being sent to a mobile phone and more frequent tracking of the pet’s position. Occasionally, companies advertise even more features for their TDs; for example, LED lights on collars for walks in the dark or the possibility of the lights glowing in a specific colour if the pet gets lost, which should help in finding the pet. Another characteristic of the analysed TDs is their water resistance. All of the TDs designed for dogs are waterproof and most of them are certified as IPX7 (immersion up to 1 m), IPX8 (immersion beyond 1 m), IP67 (dust-tight, immersion up to 1 m), or IP68 (dust-tight, immersion beyond 1 m). Even *Pawtrack* and *Cat-Control,* which are solely for cats, claim to be splash-proof.

Eight out of twenty-one of the TDs monitor the physical activity of the pet. *Whistles* can monitor different behaviour patterns, such as scratching or licking. *Findster* offers direct contact via the app to a veterinarian and charges monthly fees for this feature.

The dimensions of the TDs are between 41 mm × 29 mm × 12 mm and 96 mm × 47 mm × 45 mm. Most of the TDs’ masses range between 17 g and 50 g. Tracking units weighing more than 2% of a cat’s body mass reduce roaming behaviour [11], and some cats feel uncomfortable wearing them and attempt to remove them [12]. The TD for horses (*Hoofstep*) has a mass of 148 g, and one TD with a GNSS module (*Garmin*) that works with a frequency of 170 MHz for the far field (>10 km) has a mass of 188 g, which means that it can only be used for larger dogs.

## 3. Biological Effects of RF-EMFs and Health-Related Limit Values

**Figure 2:**
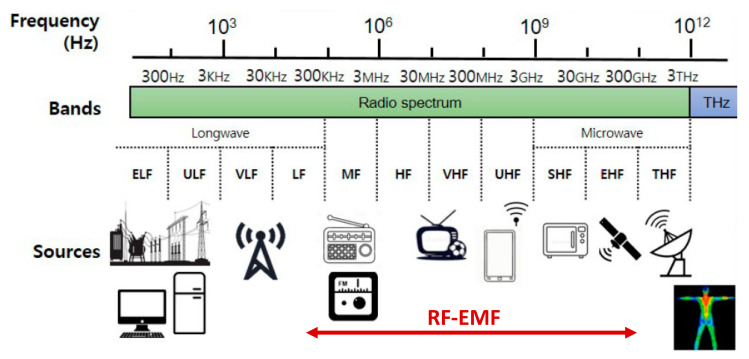
Spectrum of the radiofrequency electromagnetic field (RF-EMF) (30 kHz to 300 GHz) predominantly used for telecommunications applications (adapted from Kim et al. [13]).

The biological risks to pets of radiation in the radiofrequency (RF) range (100 kHz to 300 GHz) of the electromagnetic spectrum (Figure 2) are evaluated in this review. RF-EMFs are emitted by devices used predominantly for telecommunications, including mobile phones, and by many other sources in occupational and general environmental settings.

This frequency range causes biological and health effects that are classified as thermal and nonthermal. This classification is based predominantly on the experimental observations of the heating of biological tissue induced by this EMF. Hence, if exposure values reach or exceed the limit values for RF-EMFs, this may be detrimental to health as a consequence of energy absorption. This deterministic relationship between absorbed energy and health effects is the basis of the health-related limit values.

Thermal effects may be caused by RF-EMFs being absorbed by biological tissues as a consequence of energy absorption by tissues’ water content. The energy absorption depends on the field intensity of the EMF, the frequency of the radiation, and the electrical properties of the biological tissue. The penetration depth of an EMF is indirectly proportional to its frequency, as frequencies above 6 GHz penetrate the body up to 1 millimetre, whereas frequencies in the range of megahertz penetrate up to 30 centimetres [14]. The absorption is caused by the rotation, vibration, and movement of polar molecules and ions. This kinetic energy is converted into heat. This absorbed energy has relevant biological effects and is defined by the specific absorption rate (*SAR)*. The *SAR,* measured in watts per kilogram (W/kg), describes the absorbed RF power averaged over a certain mass of volume. This power can be averaged over the whole body or over a specific tissue or organ. The dose can be determined as the integral of the *SAR* over the exposure time [15]. Exposure to a *SAR* = 4 W/kg for 30 min results in a temperature increase of approximately 1 K in the human body. Normal muscle work equates to 3 to 5 W/kg. Additionally, a local temperature rise may result in irritation or heat damage for an organism. With regard to the effects, it is important to note that the dose, which is the product of the exposure intensity and exposure duration, normally results in accumulated damage. Hence, there are safety guidelines to prevent such impacts. If the absorbed energy is too low to cause a significant thermal effect, it is deemed to be safe [14].

In addition, there are still uncertainties about effects occurring independently of thermal effects or at very low dosages of RF-EMFs. Hence, the World Health Organisation (WHO) has stated that research on the effects initiated by RF-EMFs, such as effects on development or behaviour, reproduction, or ageing, should be given high priority [16]. There might also be nonthermal effects initiated by RF-EMFs that interfere with biological electrical activities. These effects can vary significantly with the frequency and depend on the state of an organism during the period of its exposure to RF-EMFs. Moreover, different experimental settings and conditions can result in different findings. This makes in vivo studies especially difficult, and discussions about nonthermal effects are controversial [17]. Additionally, it is a topic around which different pressure groups, such as those representing industry, politics, and science, meet, which can lead to conflicts of interest [18].

Discrepancies in the scientific evidence might be caused by the cellular mechanisms associated with RF-EMFs. These do not lead to specific impacts that are harmful to health but explain the potential risks caused by RF-EMF exposure. A primary effect caused by RF-EMFs seems to be the accumulation of reactive oxygen species, possibly even at small concentrations, which leads to biological effects [19,20,21,22,23]. However, higher levels of cellular activity or stress create significantly higher sensitivity to RF-EMFs. This leads to the possibility of higher impacts from RF-EMFs on juvenile organisms [18].

The limit values are predominantly based on thermal effects. To derive the ICNIRP limit values, an adverse health effect threshold was determined for a certain biological effect. Then, a reduction factor of 50 was applied to the threshold for the general public to cover additional uncertainties, such as age, sex, and dosimetry, and achieve a conservative limit value. These limit values are called “basic restrictions”. The reference levels derived from the basic restrictions were selected to evaluate RF-EMF exposure from TDs.

The ICNIRP reference values describe the field intensity by using the power density *PD* (W/m²) and the internal electric field strength *E* (V/m). The rate at which energy is absorbed by biological tissues is given by the specific absorption rate *SAR* (W/kg).

The ICNIRP limit values of the *SAR* distinguish between whole-body values and local areas. Using the limit values for the general public (not for occupational exposure), the *SAR* values are 0.08 W/kg for the whole body and 2.0 W/kg for local areas, such as the torso or head [14].

## 4. Exposure and Risk Assessment

The entire range of pets’ RF-EMF exposure can be divided into three categories. The first is ambient exposure, predominantly caused by radio and TV broadcasts and by base stations for mobile networks. This category is unavoidable because these RF-EMFs are ubiquitous. The second category is indoor exposure, which is caused by the intentional application of RF-EMF-emitting devices, which are mostly used indoors. The following sources were analysed in this context: digital enhanced cordless telecommunication (DECT)-based phones, wireless local area networks (WLANs), Bluetooth, and all HHDs connected to mobile networks, such as mobile phones and tablets. The third category of pet exposure is caused by wearing TDs. A risk assessment was undertaken based on the three categories of exposure. This discrimination into three categories has been used for the dose assessment of humans in terms of far-field exposure (ambient), near- to far-field expose (indoor devices), and near-field exposure by HHDs [24].

The exposure was assessed by several measures, which are summarised in Table 2. Where only one measure was presented in a paper, the corresponding measure was calculated to improve the comparability between studies. The exposure assessment was undertaken using the output power *P* (W) and the output level *P_L_* (dBm), which characterise the nominal and maximal outputs of an RF-emitting source. The output depends on the output of the power amplifier. The relationship reads as follows: *P_L_* = 10 log *P*/*P*_0_, related to *P*_0_ = 1 mW. The electric field strength *E* (V/m) and the power density *PD* (W/m^2^) describe the RF-EMF intensity, which depends on the antenna characteristics and absorption by structures such as buildings and plants. The specific absorption rate *SAR* (W/kg) gives the dose rate in the tissue of the organism, which is caused by the field intensity. Therefore, the *SAR* depends on factors such as the antenna characteristics, the geometry (distance between antenna and tissue) of the HHD, and the tissue. However, the most important predictor is the power output *P* (W) or the output power level *P_L_*. The *SAR* determines the thermal effect of an EMF and is expressed as *SAR* = *γ E*^2^/*ρ*, where *γ* is the conductivity of the tissue (S/m), *ρ* is the mass density (kg/m^3^), and *E* is the electric field strength (V/m) [14].

### 4.1. Ambient Exposure of Pets

RF-EMFs have always been present on the surface of our planet since the Sun emits solar wind that travels to the Earth. Today, the main sources of artificial RF-EMFs resulting in ambient exposure are radio and TV broadcasts and mobile networks associated with mobile base stations. Additionally, the radio applications used by the military, police, fire brigade, rescue services, and emergency medical services producing RF-EMFs must be taken into account.

The typical frequency bands used for broadcasting television and radio signals are summarised in Table 3. The output power of these EMF sources ranges from approximately 100 W up to several hundred kilowatts. These transmitters must cover large areas, whereas the base stations of mobile networks supply much smaller areas. The average distance between the base stations of mobile networks is approximately 10 km for rural areas and approximately 100 m for densely populated urban areas, with a typical transmitted power of a few to approximately 100 W.

Ambient exposure in the United States ranges from 2% below 70 mV/m (13 µW/m^2^) to 3% above 1000 mV/m (2653 µW/m^2^), with a median of 280 mV/m (208 µW/m^2^). For Germany, the change in ambient exposure due to the switch from analogue to digital broadcasting was measured as 0.3 µW/m^2^ (11 mV/m) for analogue signals and 1.9 µW/m^2^ (27 mV/m) for digital signals. For Australian adults, the overall ambient exposure was measured for 63 participants. The median personal RF-EMF exposure was determined to be 208 mV/m (115 µW/m^2^). Downlinking (file transfer from base stations for mobile networks to an HHD) contributed 40.4%, followed by broadcasting with 22.4%, uplinking (file transfer from an HHD to base stations for mobile networks) with 17.3%, and WLAN with 15.9% [26]. For 354 participants in French cities, the median downlink exposure inside dwellings close (<250 m distance) to mobile network base stations was found to be 27 mV/m (1.9 µW/m^2^), with a range of 30 mV/m (2.4 µW/m^2^) to 3580 mV/m (34,000 µW/m²). The exposure increased with the size of the city and the height of the floor of the building. The total RF-EMF exposure was determined by using the median of 44 mV/m (5.1 µW/m^2^) [27,28].

Jalilian et al. [29] conducted a systematic review of exposure in Europe in 2015. They determined the ambient exposure to RF-EMFs in homes, schools, and offices to be between 40 and 760 mV/m (4.2–1530 µW/m^2^). The mean outdoor exposure values ranged from 70 to 1270 mV/m (13–4280 µW/m^2^), with downlink signals from mobile network base stations being the most relevant contributor. The RF-EMF levels tended to increase with increasing urbanization. The levels in public transport (bus, train, and tram) and cars were between 140 and 690 mV/m (52–1260 µW/m^2^). The highest levels, up to 1970 mV/m (10,300 µW/m²), were measured in public transport stations, with downlinking as the most relevant contributor. Gajšek et al. [30] investigated EMF exposure through a comparative analysis of the results of spot or long-term measurements in the EU and indicated that the mean electric field intensity was between 80 mV/m (17 µW/m^2^) and 1800 mV/m (8600 µW/m^2^). The overwhelming majority of the mean field intensity levels were below 1000 mV/m (2650 µW/m^2^), approximately 1% were above 6000 mV/m (95,500 µW/m^2^), and approximately 0.1% were above 20,000 mV/m (1.06 × 10^6^ µW/m^2^).

A field intensity of 180 mV/m (86 µW/m^2^) was obtained for the measurement of the average exposure of humans to ambient RF-EMFs in the study by Röösli et al. [31]. This study recorded the lowest exposure indoors and the highest in public transport, such as trains, at 550 mV/m (802 µW/m^2^). The exposure inside cars was comparable to that in outdoor situations at 300 mV/m (240 µW/m^2^), while the field intensity measured in offices was higher, at 220 mV/m (128 µW/m^2^), than the lowest value of 110 mV/m (32 µW/m^2^), which was found for homes [31]. Joseph et al. [32] found median field intensities of 90 mV/m (21 µW/m^2^), 460 mV/m (561 µW/m^2^), and 740 mV/m (1450 µW/m^2^) for rural, suburban, and urban environments, respectively.

For short-term events such as fairs, the highest mean field intensity associated with base stations (downlinking) was recorded on the weekend, and it was between 1494 μW/m^2^ (317 mV/m) and 848 μW/m^2^ (565 mV/m) outside the fair area and 355 μW/m^2^ (364 mV/m) inside. After the event ended, the outside values were 556 μW/m^2^ (458 mV/m) and 144 μW/m^2^ (233 mV/m) and the intensity inside the fair area was 473 μW/m^2^ (422 mV/m). For the exposure associated with uplinking, a higher impact due to the high density was observed, with values of 28 μW/m^2^ (103 mV/m) and 98 μW/m^2^ (192 mV/m) during the fair dropping to between 5.5 μW/m^2^ (46 mV/m) and 13.6 μW/m^2^ (72 mV/m) after the fair [33].

All these values are far below those of the guidelines recommended by the ICNIRP based on the heating of tissues (41 V/m for 900 MHz, 58 V/m for 1800 MHz, and 61 V/m for 2100 MHz). A comparison of the ambient exposure values resulting from various frequency ranges and the ICNIRP reference values is available in the study by Joseph et al. [32].

Based on the field intensity, a weighted dose rate was calculated for brain tissue (thalamus, temporal lobe, and cortex). The *SAR* value was calculated to range from 10^−7^ to 10^−8^ W/kg [25] for radio and TV broadcasting. The highest variability in ambient exposure was caused by mobile base stations, which were found to cause an additional *SAR* load in the range of 10^−8^ W/kg.

The ambient exposure that pets experience can be assumed to be identical to the exposure in the human environment due to its omnipresent character.

### 4.2. Exposure by Indoor Devices

The indoor exposure that pets experience is caused by RF-emitting devices that are mostly used indoors. The following devices were included in the exposure assessment: baby surveillance, DECT base stations and mobile devices, WLAN base stations (access points and routers), Bluetooth, and personal computer peripherals. The exposure is caused by the intentional use of these devices. In this respect, the exposure can be eliminated by switching off the power supply. By reducing the operation duration and increasing the distance between the animals and the emitting devices, the exposure can be reduced considerably.

In addition to the parameters presented in Table 2, additional measures were included to characterise the exposure caused by these RF-emitting devices. The following parameters were used: the output power *P* (W); the output level *P_L_* (dBm); the effective output power *P_eff_* (W), which includes the duty factor (depending on the transfer protocol, the radio conditions, and the transmitted data (voice or uplink or downlink of data files)); and the specific absorption rate *SAR* (W/kg) (Table 4).

An important predictor for the indoor exposure of pets is the distance between an RF-emitting source and the animal. For baby surveillance and DECT devices, the impacts of the distance on the power density are summarised in Table 5. To reduce the field intensity (power density) to *PD* = 100 µW/m^2^, a safety distance was determined, which indicates the necessary distance from the RF-emitting devices to reach this intensity under the assumption of the inverse square law. This field intensity was selected according to the typical field intensity of the ambient exposure caused by radios, TVs, mobile base stations, and other omnipresent RF sources. Deviations from the inverse square law are caused by the anisotropic characteristics of the antennas of base stations. This calculation shows that, for many devices, a safety distance between 2 and 35 m is necessary to lower the exposure to such indoor devices to a level that is typical for ambient exposure (radios, TVs, and mobile network base stations).

In the following overview, the most important RF-emitting devices are analysed according to their contribution to indoor exposure.

#### 4.2.1. WLAN

A wireless local area network (WLAN) is a technology used to connect one or more devices to an access point using frequencies of approximately 2400 MHz (WLAN2) or 5200 to 5700 MHz (WLAN5). Wireless fidelity (Wi-Fi) is a special WLAN standard certified by the Institute of Electrical and Electronics Engineers IEEE-802.11 standard. The topology is either similar to a star, with one base station (access point) and several clients, or it is a mesh topology. In the star topology, the base station uses several channels to connect several devices with the base station. The effective output power of such WLAN devices depends on the number of clients and the transmission load, and the maximum output power is limited to 100 mW [37].

#### 4.2.2. Bluetooth

Bluetooth is a standard communications protocol working at 2400 MHz. The range is power class-dependent and ranges from 100 m with an output power of 100 mW (20 dBm) for class 1, 10 m with 2.5 mW (4 dBm) for class 2, 1 m with 1 mW (0 dBm) for class 3, and 0.5 m with 0.5 mW (−3 dBm) for class 4. Most applications are class 1 or 2 devices. Bluetooth has a lower data transfer capability and a lower range than WLAN. Therefore, Bluetooth is used in a single room to connect only two devices, whereas WLAN is preferred in indoor environments (apartments or houses) to connect several devices [37].

#### 4.2.3. DECT

Digital enhanced cordless telecommunication (DECT) is a communication protocol predominantly used for cordless phones; it operates at 1880–1900 MHz with a peak output power of 250 mW (24 dBm). A DECT device consists of a base station and an HHD with the same peak output power. Based on a duty factor of 4%, these devices operate with 400 μs bursts every 10 ms, resulting in an effective output power of approximately 10 mW. At a distance of 1 m, the maximum power density from the base station has been measured to be lower than 40 mW/m^2^, and the reported worst-case *SAR* was lower than 0.06 W/kg [39]. Röösli et al. [40] found a mean exposure resulting from DECT devices of 36 mV/m (3.4 µW/m^2^).

#### 4.2.4. Baby Surveillance Devices

Baby surveillance devices or baby monitors are often used to monitor children and pets. They consist of a parent unit and a baby unit, which is placed close to the baby or animal. Although a baby monitor allows bidirectional communication, it is mainly a unidirectional device from the baby unit to the parent unit [34]. Characteristic parameters, such as frequencies, duty factors, the field strength, and the *SAR*, are summarised in Table 6. The peak output power ranges between 10 mW and 500 mW [40]. In addition to technical differences, such as frequency and transmission characteristics (continuous wave with a duty factor of 100% vs. pulsed transmission with a duty factor <100%), the distance between the emitting device and the exposed body is a key feature to reduce indoor exposure [24,40]. This technology is also used for remote-controlled toys.

The exposure resulting from such indoor devices can be quantified from the exposure of single devices or by measuring the personal exposure in the indoor environment, summing up the impacts of all devices that are active. The average exposure in apartments is estimated to be between 10^−10^ and 10^−7^ W/kg [25]. These values are based on measurements of the entire indoor human environment, whereas measurement of specific exposure in the close vicinity of a single indoor device (WLAN, DECT, etc.) results in distinctly higher values, ranging between 10^−5^ and 10^−1^ W/kg (Table 4 and Table 6) [37]. Nevertheless, all the measurements are far below the ICNIRP limit values.

### 4.3. Exposure by Tracking Devices

Most of the TDs use mobile networks to transfer the tracking data, which means that the TDs work like mobile phones. A TD consists of several electronic modules and a power supply. The geographic position is determined by a GNSS module using one or more of the available GNSS signals, such as GPS and GALILEO (Table 1). The data are communicated by mobile network modules using 2G, 3G, or 4G protocols (Figure 1, pathway A) or by other radio communication frequencies that do not need a connection to a nearby mobile network base station (e.g., *PetTracer* uses a frequency of 433 MHz). These approaches are limited to the near field of the base station at home.

The exposure to RF-EMFs has been analysed for various transfer protocols (2G to 5G) and for the technical features of the corresponding HHD (Table 7) [35,36,38,41,42,43,44,45,46,47,48]. TDs work in the data transfer mode and are not used for voice calls. In general, the data traffic was found to quire higher output levels in comparison to voice calls [35,36], but these measurements were undertaken for the transfer of larger data files or video files (e.g., 40 MB with a transmission duration of approximately 30 s, [36]; video files with 640 p × 360 p, 30 fps, 3.8 min) [35]. Two different protocols are in use for the transmission of tracking data. The short message service (SMS) is used for the transfer of tracking data with a file size of 160 bytes. Later generations use the hypertext transfer protocol (HTTP) to handle the data transfer in an architectural style for an application program interface (API) called representational state transfer (REST). Compared to other data transfer protocols, REST is faster and uses less bandwidth [46,47]. Therefore, we assumed an approximately identical data transfer duration as that for SMS. Based on the data transmission bandwidth, the amount of time it takes to send a text message via SMS is approximately 0.1 s, and the output power is assumed to be 0.01 mW [48]. As the SAR values represent a mean value of 6 minutes, sending such a text message results in a much lower exposure level compared to the long-lasting transfer of large data files.

The level of exposure, which is caused by HHDs, depends strongly on the frequency and the transfer protocol used. The peak output power is reduced from 2000 mW for 2G to 200 mW for 4G and 5G. The relationship between the peak power output and the mean power for voice calls and data traffic depends predominantly on the protocol used for time slots, the bandwidth, and the frame length (e.g., 2G: bandwidth of 16.6 Hz, frame length of 4.6 ms; 4G: bandwidth of 10 kHz, frame length of 1 ms). Mobile phones using 3G and 4G techniques and protocols typically have reduced mean output power levels, depending on the quality of the connection, whereas 2G devices need much higher mean power levels, even for good quality connections [15,32,36,38] (Table 8). This results in higher exposure from the use of HHDs in rural areas with lower densities of base stations compared to urban sites [41,42,43,44].

The geometry, especially the distance between the HHD and head, but also the position, has a tremendous impact on the exposure. Even if investigations into the geometry have only been undertaken for voice calls, this effect can be transferred to data traffic as well [25,36].

Popović et al. [35] determined the mean output power (mW) for data transfer for 2G and 3G devices and Persson et al. [42] determined the *SAR* values (mW/kg) (Table 8). Due to the position of the TD on collars or harnesses, which results in a smaller distance between the TD and the head of the animal, the *SAR* values for TDs are higher compared to the human use of HHDs during data transfer.

In the RF-EMF exposure assessment, the time interval, which is used to transmit the geographic position, is a relevant parameter (Table 9) for the RF-EMF exposure level as well as for the power supply lifetime.

It is possible to transmit the position data with a constant time interval. *Pawtrack* sends the pet’s position every six minutes. *Tractive* and *Findster* offer the ability to customize the intervals for data transmission via mobile apps.

For *Simmotrade,* the intervals can be adapted between 30 s and 24 h. Most of the TDs have different modes. Some TDs can adapt the time intervals according to the physical activity of the animal. Some TDs can reduce the time interval in the case of an emergency with a setting called “live mode” or “lost mode”. The *Tractive* TDs, for instance, usually send the pet’s position every 2 to 60 min depending on its physical activity. Moreover, they have a standby mode that sends the position every ten minutes and an emergency mode that sends the position every two to three seconds. The TD for horses, *Hoofstep,* sends the position every five minutes; if the horse moves, it is sent every five seconds. These intervals are not customisable.

Some of the TDs vary the interval depending on the animal’s physical activity. For instance, *Pawfit* uses 2 to 4 h intervals if the animal rests, which is shortened to 1 to 2 min during active periods. For some TDs, these intervals can be adapted to the individual needs of pet owners, while others are default values. Most of the TDs use very short intervals ranging between 3 s (*Kippy*) and 1 min (*FI*) in emergency mode. The adaptation of the time intervals to the physical activity of the animals is one of the most effective ways to reduce RF-EMF exposure.

The TDs are normally fixed on the animals’ collar or harness, except in the case of horses, for which the TD is fixed on the horse’s head. Thus, the antenna lies near the pet’s body, especially its neck or head. This has to be considered, as the distance, next to the transmission power and duration, is essential for the organism’s exposure. The exposure resulting from such TDs might be similar to that resulting from people wearing a mobile phone in standby mode somewhere near the body all day. To reduce RF-EMF exposure, the distance between the TD and the head (brain and eyes) should be as large as possible, which means that a dog harness should be preferred over a collar.

The power output of TDs is limited in the same way as for HHDs in mobile networks. As the TDs are only used for data transfer, the field intensity depends on the quality of the reception (Table 8). The distance between the TD and the collar or harness it is mounted on can increase the *SAR* values for data transfer compared to the assumed distance of approximately 0.5 m between an HHD and the head during data transfer. Compared to the test file for human exposure, the size of the data files is much smaller and they can be transmitted in approximately 0.1 s [24,48], which considerably reduces the exposure. Compared to the average use of a mobile phone, TDs result in lower exposure.

### 4.4. Risk Assessment Regarding RF-EMF Exposure

The exposure of animals to abiotic agents such as RF-EMFs can have various effects on their health. The level of damage depends on the exposure to the agent and the related dose–response function. RF-EMF exposure limit values have been established by the ICNIRP and provide a high level of protection for all humans against substantial adverse health effects from exposure to both short- and long-term, continuous and discontinuous RF-EMFs. To transfer these findings to animals, we assumed that animals show a biological response to exposure that is equivalent to that of humans. As many experiments are performed on animals and the findings transferred to humans, this step was reversed to assess the biological effects of RF-EMFs on pets. If the *SAR* values do not exceed the ICNIRP thresholds and no biological effects have been detected after RF-EMF exposure in laboratory animals, which are usually smaller mammals than our companion animals, then similar or even lower *SAR* values can be expected for companion animals [14,25,39,49]. Furthermore, the number of studies about effects from RF-EMFs on companion animals, like cats or dogs, is limited. Moreover, these studies usually address highly specific cellular processes, which are not decisive for the health assessment of pets [50,51]. The ICNIRP reference levels for humans were here applied to animals.

The exposure of pets was divided into the following three categories: (1) ambient exposure (e.g., radio and TV broadcasts, base stations of mobile networks), which is omnipresent and is closely related to a particular site; (2) indoor exposure, which depends on the intentional use of DECT, WLAN, and other RF-emitting devices mostly used indoors; and (3) intentional exposure due to the use of TDs.

Horses, which are normally kept indoors in stables and outdoors in rural environments, might have lower exposure levels due to the lower field intensity in rural environments [32]. The same conclusion can be drawn with regard to free-roaming cats living in rural environments.

Additionally, the use of mobile phone communication devices in close proximity to animals could have an impact on their RF-EMF exposure, as 35% of the indoor exposure that humans experience comes from mobile phone usage in the near surroundings [31]. The same concept can be applied to cats, dogs, and horses. The variability of the indoor dose, which is caused by the variation in the exposure time and the distance from RF-transmitting devices, is discussed in detail with regard to humans by Van Wel et al. [24].

Christ et al. [52] showed that for children, exposure related to the use of mobile phones can be significantly higher in certain subregions of the brain (the cortex, hippocampus, and hypothalamus) and in the eye due to the closer proximity of HHDs to these tissues, as well as in bone marrow in the skull as a result of its significantly high conductivity. These findings can be transferred to juvenile pets, even if the geometry of the TD and the head of the animal is different due to the use of collars to mount the TD. For the hippocampus, in children, *SAR* values increased by a factor of 2 (3 dB) and a factor of 1.6 (2 dB) at 900 MHz and 1800 MHz, respectively. For the eyes of children, the *SAR* was increased by a factor between 4 and 10 (6–10 dB), with the frequency showing no impact. This shows that the risk for juvenile pets is also higher, and this is caused by the geometry (smaller distance), the conductivity of juvenile bones, and the frequency of the TD.

The data transmission mode of mobile phones was tested using the long-lasting transmission of data files (e.g., 40 MB data file with a duration of approximately 30 s, [36]; video files (640 p × 360 p, 30 fps, 3.8 min) [35]). Based on the bandwidth of the data transmission, the time taken to send an SMS message is approximately 0.1 s, and the output power is assumed to be 0.01 mW. Exposure associated with the transmission protocols of later generations of mobile networks (3G, 4G, etc.) may result in comparable exposure levels. This means that the corresponding risk for pets wearing a TD is much lower compared to the use of mobile phones in voice mode for a person [48].

Some meta-studies have analysed the risk of cancer in humans due to the use of mobile phones. The Scientific Committee on Emerging and Newly Identified Health Risks (SCENIHR) [39] stated that there is little evidence that moderate use of mobile phones is associated with any cancer in the head and neck region in humans. The International Agency for Research on Cancer (IARC) [25] found *limited evidence* in humans for the carcinogenicity of radiofrequency radiation. Positive associations have been observed between exposure to radiofrequency radiation from mobile phones and glioma and acoustic neuroma. RF-EMFs have been categorised as agents that are possibly carcinogenic to humans. The ICNIRP [14] concluded that the only substantiated adverse health effects caused by exposure to RF-EMFs are nerve stimulation, changes in the permeability of cell membranes, and temperature elevation effects. There is no evidence of adverse health effects at exposure levels below the restriction levels of the ICNIRP.

In addition to the risks resulting from RF-EMF exposure, the risks of wearing a collar [53,54,55] or harness have to be taken into account. Studies using research-specific TDs and other studies using TDs mounted on breakaway collars or harnesses equipped with breakaway clips did not report problems attributed to collar or harness usage in cats [56,57,58,59,60]. The proper use of collars/harnesses with breakaway mechanisms is of utmost importance for unsupervised animals, as one study reported the strangulation of three cats (9%; *n* = 34) using adjustable metal collars fastened with a bolt and nut almost immediately after collar placement [61]. Independently of the collar type, the risk of entrapment and injury for cats cannot be fully prevented; even with breakaway collars, severe injuries can occur [62,63]. One important aspect to reduce risk is a good collar fit, meaning that the collar is neither too loose nor too tight [54,55,63].

## 5. Conclusions and Recommendations

The examination of the RF-EMF exposure of pets and the related risk assessment show that the sum of the values for all three categories of exposure (ambient and unintended exposure, intended exposure to indoor RF-emitting devices, and intended exposure due to TDs) lies distinctly below the ICNIRP reference levels. This means that adverse health effects on animals from exposure to RF-EMFs can be largely excluded.

Even if the RF-EMF exposure level lies below the ICNIRP limit values, the following measures could be taken to reduce the exposure and to improve the efficacy of using TDs:

- The exposure from RF-emitting indoor devices can be eliminated or reduced by switching off the power supply or limiting the operating time of technical devices;
- A sufficient distance between RF-emitting indoor devices and animals should be maintained. This should especially be considered with regard to the resting areas of animals;
- A TD should only be applied in those periods in which the pets have outdoor access and may run away;
- For juvenile animals, a higher degree of caution regarding indoor exposure and the use of TDs should be considered because the same exposure to a certain level of field intensity can result in a higher dose rate compared to adult animals;
- The TD should be selected according to the transmission protocols used. This means that newer technologies (3G or 4G) should be preferred because these transfer protocols reduce the overall output power more effectively;
- The TD should offer the possibility of configuring the time interval with which the geographical position is sent, thus reducing exposure by reducing the data transfer. Some TDs adapt the intervals based on animals’ physical activity (moving vs. resting);
- To reduce the exposure to RF-EMFs, a harness can be used instead of a collar to attach the TD. With a harness, the distance between the head (the brain and eyes) and the TD can be increased and thus the dose rate (*SAR* value) can be considerably reduced. However, the risks of harness use for cats have not been investigated thus far;
- The name of the pet’s register and the pet’s ID number should always be available on the collar/harness of a dog or cat in case the tracking device is out of order (e.g., low power supply, no connection to transmit the position data);
- Animals should be trained via positive reinforcement to tolerate collars/harnesses and mounted objects such as TDs.

## Figures and Tables

**Table 1:** Technical features of the investigated tracking devices.

Company	Species	Tracking Technology	Dimensions (mm^3^)	Mass (g)	Communication Technology (Far Field)	Communication Technology (Near Field)	Power Supply
PAJ GPS; Pet Finder PAJ GPS; ALLROUND Tracker	Dogs, cats Horses	GPS GPS	57 × 32 × 16 106 × 63 × 22	28 140	2G 2G	-	3.7 V 500 mAh Li-ion; standby: 2–3 d; everyday tracking: 1–2 d standby: 20–60 d
Simmotrade; TKSTAR 911	Cats, dogs	GPS, LBS	62 × 30 × 18	33	2G	-	3.7 V 500 mAh Li-ion; standby: 8 d
Simmotrade; TK909	Dogs	GPS, LBS	70 × 37 × 20	44	2G	-	3.7 V 600 mAh Li-ion, standby: 12.5 d
Kippy; Kippy Vita S Black Guardian	Cats, dogs	GPS	61 × 44 × 27	48	2G	-	Up to 7 d
Tracker ID; NX-4440-919	Dogs, other pets	GPS, LBS	52 × 39 × 17	33	2G	-	420 mAh LiPo
Pawtrack; Pawtrack GPS Cat Collar	Cats	GPS, GALILEO	Collars in three different sizes	35	2G	WLAN	2 d 2 h to recharge
FI; FI Series 2	Dogs	GPS	Collars for circumferences of the neck >29 cm	-	4G	Bluetooth, WLAN	3 m to 3 w and 2 d in lost-dog mode
Pawfit; Pawfit 2	Cats, dogs	GPS, LBS	50 × 35 × 15	30	2G	WLAN	Up to 6 d
Tractive; GPS Dog 4	Dogs	GPS	71 × 28 × 17	35	2G	-	Up to 5 d
Tractive; GPS Cat Tracker	Cats	GPS	72 × 29 × 16	28	2G	-	2–5 d
Tail It; Tail It pet	Cats, dogs	GPS, LBS	41 × 29 × 12	23	2G	WLAN	520 mAh lithium, 14 d in standby
FirBark; FitBark GPS	Dogs	GPS, LBS	NA	17	4G	Bluetooth, WLAN	10–20 d
Whistle; Whistle Go Explore	Dogs, other pets >3.6 kg	GPS, LBS	36 × 46 × 18	28	4G	WLAN	Up to 20 d
Petfon; Pet GPS-Tracker	Dogs	GPS, GLONASS	42 × 42 × 18	24	No SIM card 916 MHz+ PetFon Mash <10 km	916 MHz (<5 km)	Polymer lithium; up to 8 h
Findster; Findster Duo+	Cats, dogs	GPS	50 × 50 × 13	21	-	900 MHz (4.8 km)	12 h to 7 d
HoofStep	Horses	GPS	96 × 47 × 45	149	-	2400 MHz (1 km)	21 d (3 h charging)
Cat-Control; Cat-Control Katzenpeilsender	Cats	-	30 (diameter) × 5	5	-	Transmitter + loop antenna 2400 MHz (120 m)	Up to 5 m
Pet Tracer; PetTracer Set EU	Cats	GPS	Collars for neck circumferences >21.5 cm	34	-	Transmitter + loop antenna 433 MHz (1.6 km)	Up to 30 d
Garmin; Atemos 50/K5 System	Dogs	GPS, GLONASS	89 × 44 × 47 collars for neck circumferences >24 cm	188	No SIM card 170 MHz <10 km	-	Lithium ion 20–40 h
Telekom, Alcatel Combi Protect	Dogs	GPS, GLONASS	42 × 42 × 16.3	33	2G	-	460 mAh Li-ion Up to 4 d

Abbreviations: LBS, location-based service; GPS, Global Positioning System.

**Table 2:** Parameters to characterise the exposure to RF-EMFs: output power of the EMF-emitting sources, the field intensity, and the dose rate [14].

Parameters for the Exposure Assessment		Equation
Emission	Output power *P* (W) or output power level *P_L_* (dBm), related to *P*_0_ = 1 mW	*P_L_* = 10 log *P/P*_0_
Field intensity	Electric field strength *E* (V/m) and power density *PD* (W/m^2^)	*PD* = *E*²/377 Ω
Dose rate	Specific absorption rate *SAR* (W/kg)	*SAR* = *γ E*^2^/*ρ*

**Table 3:** Frequency bands for television and radio signals [25].

Designation	Frequency (Mhz)	Application
Long-wave	0.146–0.284	AM radio
Medium-wave	0.527–1.607	AM radio
Short-wave	3.9–26	International radio
UHF	470–854	Analogue and digital TV
VHF (band II)	88–108	FM radio
VHF (band III)	174–226	DAB and analogue/digital TV

Abbreviations: UHF, ultra high frequency; VHF, very high frequency, AM, amplitude modulation; FM, frequency modulation; DAB, digital audio broadcasting.

**Table 4:** Technical characteristics of RF-emitting indoor devices: peak output given in mW and dBm, the effective output level taking into account the duty factor, and the corresponding *SAR* values.

Parameters	WLAN	DECT	Baby Surveillance	Bluetooth	Ref.
Frequency	2400	1800	400/2450	2400	
Peak output power (mW/dBm)	100/20	250/24	500/27	100/20	[34]
Effective output power level (dBm)	Mean 19.6 Rural areas: 90-p: 33 10-p: 5	Mean 18 to 24	27 (400 MHz) −7 (1900 MHz) −6 to 4.2 (2400 MHz)		[35]
*SAR* (µW/kg)	60–810	13 to 27	10 to 77	466	[36]
	105–136				[37,38]
			40 to −370		[35]

Abbreviations: 90-p, 90th percentile; 10-p, 10th percentile.

**Table 5:** Attenuation of the power density *PD* (µW/m^2^) according to the distance between the source and the animal. A safety distance was determined in order to reduce the power density to *PD* = 100 µW/m^2^, which was assumed to be a typical value for ambient exposure. For the DECT devices, four units were measured.

Power Density *PD* (µW/m^2^) and the Distance from the Source (m)		Frequency (MHz)	Safety Distance (m)	Source and Reference
192,000 (0.2 m)	27,000 (1 m)	863	21	Baby surveillance [36]
350,000 (0.2 m)	22,300 (1 m)	1900	20	
3151 (1 m)	446 (3 m)	446	7	Baby surveillance [34]
537 (1 m)	52 (3 m)	864	2	
424 (1 m)	32 (3 m)	2450	2	
34,570 (1 m)	4436 (3 m)	1900	21	DECT [34]
4079 (1 m)	514 (3 m)		7	
19,190 (1 m)	5968 (3 m)		35	
9880 (1 m)	1573 (3 m)		13	

**Table 6:** Characteristic values of baby-surveillance devices: frequency, duty factor (%), electric field strength *E* (V/m) measured at a distance of 50 cm, *SAR* (W/kg) measured close to the baby unit, and ICNIRP limit values ( [39]).

Frequency (Mhz)	ICNIRP Limit Values	Duty Factor (%)	Electric Field Strength *E* (mV/m) and Power Density *PD* (µW/m^2^)	*SAR*
	Power Density *PD* (W/m^2^)			(W/kg)
446	2.23	100	550/1500	0.04 to 0.37
864	4.32	100	802/6000	
1900	9.55	4	550/880	0.03 to 0.15
			802/2100	
2400	10	5–53	220/1600	0.09 to 0.21
			128/6800	

**Table 7:** Characteristics of handheld devices (HHDs) for various transfer protocols (2G to 5G) and types of data traffic.

	2G GSM900	2G GSM1800	3G GPRS	3G UMTS/WCDMA	4G LTE	5G	Comment/ Reference
Frequency	900	1800	900–1900	1900–2100	800–2600	700–3800	
Peak output power (mW/dBm)	2000/33	1000/30	250/24	250/24	200/23	200/23	[41,42,43]
Mean output power level (dBm)	Mean: 19.6 Rural— 90-p: 33 dBm 10-p: 5 dBm	Mean: 18–24	Median: 4.0	Median: −14 90-p: −1.5 Suburban— Median: −17 90-p: −4.0 Voice/rural— Median: −9.0 95-p: 11.9 Data/rural— Median: −2.6 95-p: 17.4	Rural— Median: −6.3 95-p: 6.4 Suburban— Median −9.9 95-p: 1.2	Median: 3.0 95-p: 12	[43,44,45,46,47,48]
Electric field strength *E* (V/m)	18.5–209	18.5–209		0.98–68.5	3.03–69.3		4 MB and 30 s [42]
Maximum and mean contributions of the field strength to the total field strength E_max_/E_mean_ (%)	100/53	87/15		90/6	23/0.4		[34]
SAR (µW/kg)	90–2400			15–550			[38]

Abbreviations: 95-p, 95th percentile; 90-p, 90th percentile; 10-p, 10th percentile.

**Table 8:** Mean output power (mW) and *SAR* values (mW/kg) for 2G and 3G HHDs for data uploading depending on the quality of the connection.

Quality of the Connection	2G (900 MHz)	3G (2100 MHz)	Reference
	**Mean Output Power (mW)**		
High	69	1.8	[38]
Low	1800	170	
	***SAR*** **(mW/kg)**		
High	0.091	0.015	[45]
Medium	1.978	0.88	
Low	2.399	0.548	

**Table 9:** Intervals for sending the tracking device position data.

Tracking Devices	Customisable	Default Intervals	Emergency-Mode
Paj GPS	NA	NA	NA
Simmotrade	30 s to 24 h		
Kippy	Update by request		3 s
Tracker ID	>1 min		
Pawtrack		6 min	
FI		2–3 min	1 min
Pawfit		5 s to 4 h 2–4 h resting 1–2 min walking	5 s
Tractive		60 min resting 10 min moving	2–3 s
Tail It Pet	Update by request		5 s
FitBark		1 min	1 min
Whistle	3 or 6 min		15 s
PetFon	10, 20, or 60 s		
Findster	Update by request	10 s	
Hoofstep		5 min resting 5 s moving	
Cat-Control	NA	NA	NA
PetTracer	1, 3, or 15 min		15 s
Garmin	>5 s		

NA, no data available.

## Abbreviations

DECT	digital enhanced cordless telecommunication
GNSS	global navigation satellite system
GPS	Global Positioning System
HHD	handheld device
IARC	International Agency for Research on Cancer
ICNIRP	International Commission on Non-Ionising Radiation Protection
LBS	location-based service
PD	power density
RF-EMF	radiofrequency electromagnetic field
RFID	radiofrequency identification
SAR	specific absorption rate
SCENIHR	Scientific Committee on Emerging and Newly Identified Health Risks
TD	tracking device
WHO	World Health Organisation
WLAN	wireless local area network
